# Implementation and Audit of Mainstream Genetic Testing Within a High‐Volume UK Breast Unit for Pathogenic Variations Associated With Breast Cancer Using the R208 and R444.1 National Test Directory Criterion

**DOI:** 10.1155/tbj/2657384

**Published:** 2026-07-27

**Authors:** Soraya Conroy, Emma Keane, Bushra Rehman, Eleanor Wyborn, Catherine Brown, Lamb Chen, Josh Chatten, Elijah Durston-Rand, Blaise Sheehan, Elizabeth Ferguson, Tracie Miles, Emma Beck, Alison Hunter-Smith, Christiana Laban, Katherine Smith, Diana Dalgliesh, Rebecca Bowen, Nicola Laurence

**Affiliations:** ^1^ Breast Unit, Royal United Hospital, Bath, UK; ^2^ Computer Science Department, University of Bristol, Bristol, UK, bristol.ac.uk; ^3^ Medical School, University of Bristol, Bristol, UK, bristol.ac.uk

## Abstract

**Introduction:**

It is estimated that 5%–10% of patients who develop breast cancer have a causative inherited pathogenic or likely pathogenic variant (P/LP variant). In 2020, the UK National Test Directory published criteria for mainstream genetic testing for breast cancer patients (R208) and, subsequently, eligibility criteria to determine which patients are eligible for gene testing and PARP inhibitor treatment (R444.1).

**Methods:**

Using the clearly defined criteria from NHS genomics, eligibility for testing under R208/R444.1 was determined for all patients diagnosed with breast cancer between March 2021 and March 2025. Eligible patients were offered genetic testing. Incidence of P/LP variants and impact on treatment were examined.

**Results:**

A total of 1812 patients had new DCIS/invasive breast cancer diagnoses, 255 were eligible for testing and 196 consented. Of these, 28 patients (14.3%) had a P/LP variant. Eight of these patients were eligible only using family history criteria. Twenty‐one patients were eligible for breast conservation surgery. Preoperative genetic results were available for 13: eight patients opted for bilateral mastectomy rather than breast conservation. Where results were available postoperatively, three of eight patients who had breast conservation are planning bilateral risk reducing surgery. Three women newly diagnosed with a BRCA variant received a PARP inhibitor. Eligibility assessment for testing was time‐consuming for trained clinicians.

**Conclusion:**

14.3% of patients eligible and tested for a breast cancer–related hereditary P/LP variant were positive, which aligns with the expected number predicted by Genomics England. Family history scoring is an important element. Positive results were associated with changes in surgical decision‐making for 14 women and enabled 3 patients to receive a PARP inhibitor.

## 1. Introduction

With approximately 2.3 million new breast cancer diagnoses each year, it has one of the highest hereditary causes of all cancers. 5%–10% of those diagnosed are thought to harbor a pathogenic or likely pathogenic variant (P/LP variant), increasing their susceptibility to the disease [1, 2]. Mainstream genetic testing for breast cancer was introduced in the UK in 2018 under clinical indications described in the National Genomic Test Directory (R208) to streamline access [3, 4]. This set out standardized criteria and a clinical pathway for genetic testing across the country, enabling nongenetic specialists including oncologists, breast clinicians, breast surgeons, and clinical nurse specialists to counsel and offer genetic tests to eligible patients [5, 6]. In line with NICE guidance (CG164), these criteria were designed to identify those patients with a ≥ 10% probability of carrying a P/LP variant [7].

In July 2023, following implementation of R208, the R444.1 panel was also offered to patients who may benefit from treatment with a PARP (poly‐ADP ribose polymerase) inhibitor if found to have a BRCA pathogenic variant. This set of complex criteria is based on postoperative pathological factors [6]. Initially, offering the full panel of genes, this was changed to BRCA1, BRCA2, and PALB2 only [8]. The R208 eligibility criteria were also expanded, increasing the age cutoff for some subgroups [6].

Implementation of mainstream testing requires several processes. These include a process to assess eligibility of each patient diagnosed with invasive breast cancer or high‐grade DCIS as well as a program of training for clinicians to counsel on the risks and benefits of genetic testing, obtain informed consent and deliver the results [9, 10].

**FIGURE 1:**
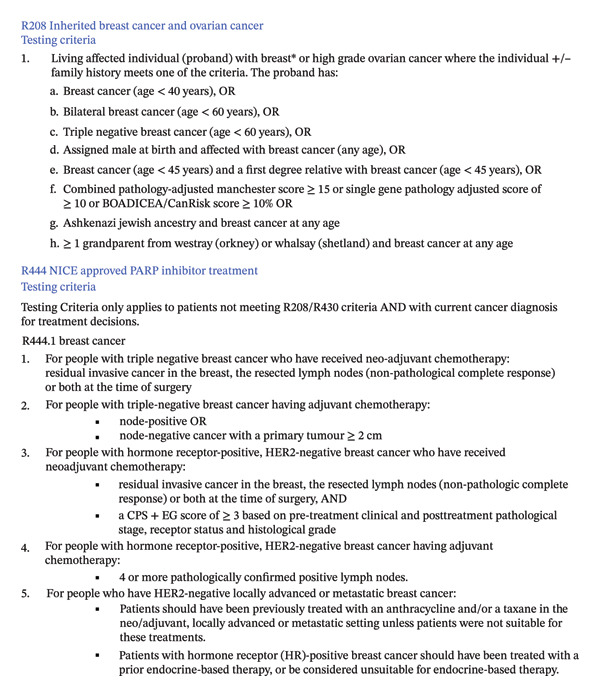
R208 and R444.1 testing criteria—National Genomic Test Directory, testing criteria for rare and inherited disease (version 8.1 July 2025). *Source*: NHS England National Genomic Test Directory. https://www.england.nhs.uk/wp-content/uploads/2018/08/rare-and-inherited-disease-eligibility-criteria-V8.1.pdf [6].

**FIGURE A1:**
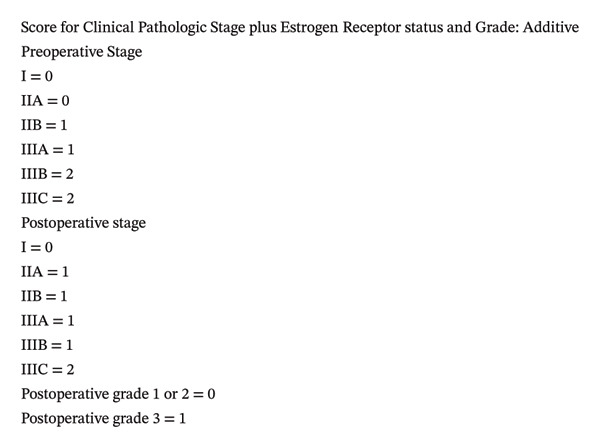
Score for clinical pathologic stage plus estrogen receptor status and grade: additive.

Some of the R208 eligibility criteria are categorical data such as age at diagnosis, triple‐negative receptor status, and bilateral disease, which are readily and easily identified. However, eligibility based on family history requires more complex and detailed information for calculations to be made based on both the affected family member/s and specific tumor factors (Fig 1) [11–14]. The eligibility criteria for R444.1 are complex and challenging to assess within the multidisciplinary team (MDT) meeting. This included calculation of the CPS (Clinical Pathologic Stage) + EG (Estrogen receptor status and Grade) score (Figure A1). It is important to ensure all patients who are eligible receive the opportunity for testing and access to PARPi where appropriate (Figure 1). There is an argument to offer genetic testing to all patients who have a breast cancer diagnosis [15]. However, testing all would mean a significant amount of time spent counseling, taking blood, testing, and delivering results when just a small proportion of patients outside of the R208 criteria will harbor a P/LP variant. Clinical time might be better spent empowering patients found to have a P/LP variant to make well‐informed choices as regard to their treatment.

We implemented and prospectively audited the pathway to establish which of our patients were eligible for genetic testing under R208 criteria. We determined what proportion of those tested were found to have a P/LP variant and if this aligned to > 10%, predicted by NHS genomics. Furthermore, which of the R208 eligibility criteria were met and the impact this had on patient decision making for surgical treatment. Finally, we looked to see how many patients had tested under R444.1 and which received a PARPi.

## 2. Method

Following published guidance and clinician attendance at an NHS genomics online education program, the standardized protocol was implemented. While not independently checked, the protocol followed guidance from South West genomics [5]. All newly referred patients to the breast unit at the Royal United Hospital (RUH), Bath, were sent a questionnaire to complete prior to their initial appointment. Questions were asked about their presentation, as well as those relevant to the Manchester scoring system to assess eligibility for testing. This information was available in the electronic records.

Each week, responsible clinicians reviewed all patients with a new breast cancer diagnosis from the MDT meeting list. Clinical parameters and family history data were assessed for eligibility for testing under R208 as detailed in Figure 1. Family history eligibility was assessed with the Manchester score or the CanRisk score.

The Manchester score is a tool used to assess a patient’s family history of cancer and correlates with the likelihood of an underlying P/LP variant in BRCA1 or BRCA2 being present [12, 13]. Manchester scores were calculated for those patients who did not fulfill other eligibility criteria. Where patients had a family history of breast, ovarian, pancreatic, or prostate cancer but did not reach a Manchester score of 15, CanRisk assessments were undertaken. CanRisk is a web‐based tool to predict each individual’s risk of developing breast, ovarian, and prostate cancers, as well as the probability of having a P/LP variant in high‐ and moderate‐risk genes [11, 14, 16, 17].

Postoperative patient data were reviewed for assessment of eligibility for R444.1/PARPi based on diagnostic and postoperative histology (Figure 1).

All breast cancer patients were discussed at the MDT meeting, and those eligible for R208 testing were recorded. The patient was counseled, either by the trained consultant or by the cancer nurse specialists.

Patients who consented to testing had blood sent to the Genomic Hub laboratory in Bristol. Table 1 illustrates the genes tested for under the R208 and R444.1 criteria. Results were returned to the patients within 2–12 weeks.

**TABLE 1:** Genes tested under R208 and R444.1.

	R208	R444.1
Genes Tested	BRCA1	BRCA1
	BRCA2	BRCA2
	PALB2	PALB2
	CHEK2	
	ATM	
	RAD51C	
	RAD51D	

All patients who were found to have a P/LP variant were referred to the Clinical Genetics Rapid Access Cancer Clinic. Here, patients received further counseling on cancer risks, enhanced screening, risk‐reduction surgery, and implications for family members.

Those patients with a high‐risk family history or those < 30 years who were not found to have a P/LP variant were referred to the clinical genetics team for further counseling about their risk, relative to the general population and consideration of further testing. Those with a reported variation of unknown significance (VUS) would have been referred on to genetics.

An audit of this prospectively implemented pathway was registered with the hospital audit department (audit code: BREAST/CA/2025‐26/02). Ethical approval was not required as this was an audit/service evaluation. The audit was carried out to ensure that the assumption from NHS genomics, that approximately 10% of patients tested would have a P/LP variant, was true in our unit. Data were included for consecutive patients diagnosed with breast cancer between March 2021 and 2025.

Data to identify all patients eligible for testing were determined from the patient record. Eligibility assessment followed a standardized protocol as recommended by NHS genomics [6]. For those eligible patients, records were analyzed to determine reasons for eligibility and if the patient was tested. If not tested, documentation was scrutinized to determine why. If there was no reason recorded as to why the patient was not tested, the assumption was made that genetic testing was not discussed as a clinical error. There would be the possibility that the patient declined, but unless documented, these patients were recorded as not discussed. For those tested, results were recorded. Patients with each P/LP variant were recorded. The medical and surgical choices (including breast conservation versus mastectomy and eligibility for PARPi) were recorded.

During this time frame, eight patients, already known to have a P/LP variant but declined risk reduction surgery, were diagnosed with breast cancer. They were excluded from the numbers of patients diagnosed through mainstream testing.

## 3. Results

**FIGURE 2:**
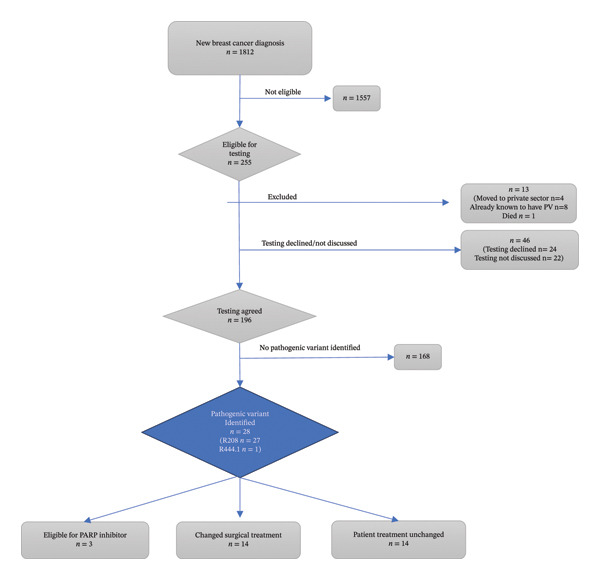
Number of patients eligible, tested and found to have a P/LP variant and impact on treatment (PARP = poly‐ADP ribose polymerase).

Between March 2021 and March 2025, 1812 patients received a new breast cancer diagnosis. Two hundred and fifty‐five were eligible for genetic testing based on the R208 or R444.1 criteria (Figure 2). Thirteen patients were excluded as their care moved to the private sector, died prior to discussion, or already had a known P/LP variant. Twenty‐four patients declined testing. As part of the informed consent process, patients were informed that if testing is initially declined, the patient could reconsider this option. Reasons for declining testing were not always recorded. If recorded, the most common reason was that they would consider it in the future. A further 22 patients were eligible but not tested; it is presumed that testing was not discussed but this information was limited. One hundred and ninety‐six patients underwent genetic testing. Of the 191 patients who were tested under R208, 27 were found to have a P/LP variant. Three of those were eligible for a PARPi. Of the five patients tested under R444.1, one was found to have a P/LP variant. Supporting Table 1 shows the specific variations identified.

14.3% of those tested had a P/LP variant, resulting in an overall rate of all patients diagnosed with breast cancer during the study period of 1.5%.

No patients had a reported VUS. With mainstream testing, patients with a variant of uncertain clinical significance (VUS) are not reported back as such unless this approaches the likely pathogenic classification boundary.

**FIGURE 3:**
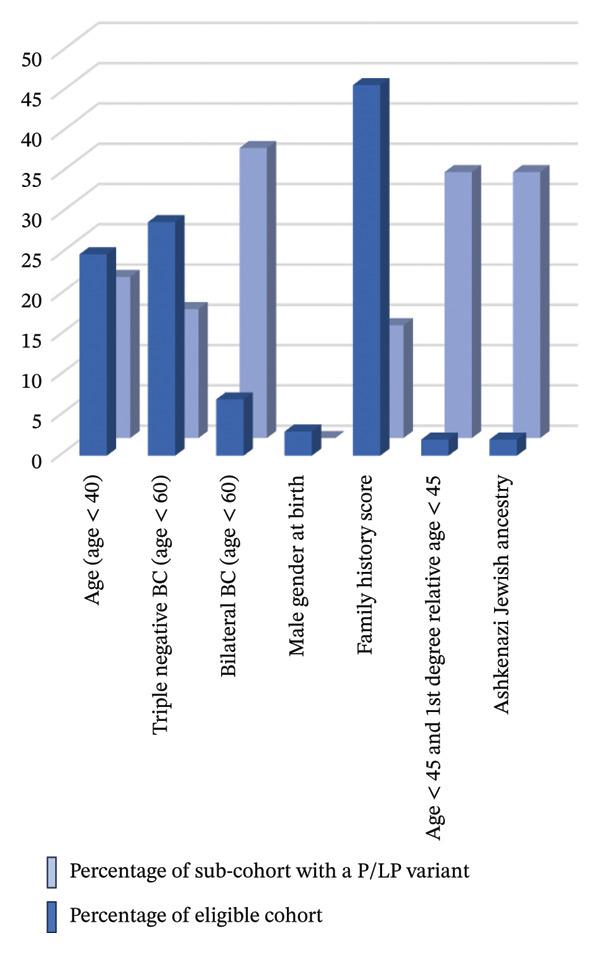
Percentage of patients eligible for testing under each R208 criterion and the percent of those found to have a P/LP variant (BC =breast cancer, age = years).

Figure 3 highlights the percentage of patients eligible for testing under each R208, and the percent was found to have a P/LP variant. Those who fulfilled more than one criterion for testing were recorded more than once, although complex family history scores may not have been determined if patients had met a simpler R208 criterion.

The most common genetic variants noted were BRCA1 and CHEK2. Eight patients identified as having a P/LP variant were eligible and offered testing based on their family history score alone.

### 3.1. Patients Eligible for Breast Conservation Breast Cancer Surgery (Table 2)

Of the 27 patients found to have a P/LP variant through R208, 21 patients were eligible for breast conservation surgery. Eligibility for R444.1 is determined postoperatively.

**TABLE 2:** Patient characteristics, mutation status, surgical decision‐making, and PARP inhibitor eligibility in the initial treatment pathway.

Pathogenic/likely pathogenic variation	Age at diagnosis (years)	Eligibility checklist met: (R208/R444.1) (age in years)	Result known at time of surgical phase of treatment	Breast conservation surgery	Postoperative stage ER & Her2	Mastectomy therapeutic side	Mastectomy contralateral side	PARP inhibitor prescribed
BRCA1	37	BC (age < 40) Bilateral BC (age < 60) Triple negative BC (age < 60)	Yes	No, was eligible	ypT0N0M0 ER 0/8 Her2 –ve	Yes	Yes, therapeutic (bilateral cancer)	No

BRCA1	44	Ashkenazi Jewish ancestry	Yes	No, was eligible	ypT2N1a ER 8/8 Her2 −ve	Yes	Yes, RRM at time of cancer surgery	No

BRCA1	28	BC (age < 40)	Yes	No, was eligible	ypT0N1a ER 3/8 Her2 –ve	Yes	Yes, RRM at time of cancer surgery	Eligible, prescribed

BRCA2	66	Family history score	Yes	No, was eligible	ypT0N0 ER 0/8 Her2 −ve	Yes	Yes, RRM at time of cancer surgery	No

PALB2	50	Triple negative BC (age < 60) Family history score	Yes	No, was eligible	ypT0N0 ER 2/8 Her2 −ve	Yes	Planned at a later date	N/A

CHEK2	39	BC (age < 40) Triple negative BC (age < 60)	Yes	No, was eligible	pT2N1(mic) ER 0/8 Her2 −ve	Yes	Yes, RRM at time of cancer surgery	N/A

CHEK2	48	Bilateral BC (age < 60) Family history score	Yes	No, was eligible on one side only	ypT3N1a ypT0N0 ER 8/8 Her2 +ve bilaterally	Yes	Yes, therapeutic (bilateral cancer)	N/A

ATM	34	BC (age < 40)	Yes	No, was eligible	ypT1N0M0 ER 8/8 Her2 –ve	Yes	Yes, RRM at time of cancer surgery	N/A

BRCA1	45	Triple negative BC (age < 60)	Yes	Yes	ypT0N1a ER 0/8 Her2 –ve	Planned for bilateral DIEP when BMI lower	Planned at a later date	Eligible, prescribed

PALB2	37	BC (age < 40)	Yes	Yes	ypTisN0 ER 3/8 Her2 −ve	No	No	N/A

CHEK2	56	Triple negative BC (age < 60) Bilateral BC (age < 60)	Yes	Yes	pT1N0 ER 0/8 Her2 –ve	No	Previous therapeutic mastectomy	N/A

CHEK2	36	BC (age < 40) Family history score BC (age < 45) + first degree relative with BC (age < 45)	Yes	Yes	ypT1c N1a ER 6/8 Her2 +ve	Planned for bilateral DIEP when BMI lower	Planned at a later date	N/A

CHEK2	45	Family history score	Yes	Yes	ypT0N0 ER 5/8 Her2 +ve	No	No	N/A

PALB2	79	Bilateral BC (age < 60) Family history score	Yes	No, not eligible	pT1N0M0 ER 2/8 Her2 −ve	Yes	No but elderly (late 70s)	N/A

BRCA1	68	Family history score	No	Yes	pT2N1a ER 8/8 Her2 −ve	Planned RRM but became metastatic	Planned RRM but became metastatic	No

BRCA1	37	BC (age < 40)	No	Yes	pT2N0 ER 8 Her2 1+	Planned at a later date	Planned at a later date	No

BRCA2	66	Family history score	No	Yes	pT1N0 ER 8 Her2 0	Planned at a later date	Planned at a later date	No

BRCA2	48	Triple negative BC (age < 60)	No	Yes	ypT0N0 ER 0 Her2 0	Planned at a later date	Planned at a later date	No

BRCA2	59	Family history score	No	Yes	pT1N0 ER 8/8 Her2 −ve	No	No	No

PALB2	70	R444.1	No	Yes	pT1N1 ER 0/8 Her2 1+	No	No	N/A

CHEK2	54	Family history score	No	Yes	pT1N0 ER 5/8 Her2 −ve	No	No	N/A

RAD51D	49	Triple negative BC (age < 60)	No	Yes	pT2N0M0 ER 0/8 Her2 −ve	No	No	N/A

BRCA1	38	BC (age < 40) Family history score	No	No, not eligible	ypT2N2a ER 7/8 Her2 1+	Yes	Planned at a later date	Eligible, prescribed

BRCA2	47	Bilateral BC (age < 60)	No	No, not eligible	pT3 N3a ER 8/8 Her2 −ve	Yes	Previous therapeutic mastectomy	Eligible: 2021 not offered

BRCA2	69	Family history score	No	No, not eligible	ypT2Nx (inoperable) ER3 Her2 −ve	Yes	Previous therapeutic mastectomy	No

CHEK2	32	BC (age < 40)	No	No, not eligible	pT2N1a ER 8/8 Her2 +ve	Yes	No	N/A

BRCA1	43	BC (age < 40) Bilateral BC (age < 60) Triple negative BC (age < 60)	N/A: metastatic		M1			No

CHEK2	51	Family history score	N/A metastatic		M1			N/A

*Note:* DIEP, deep inferior epigastric artery perforator; Her2, human epidermal growth factor receptor 2.

Abbreviations: ANC, axillary node clearance; BC, breast cancer; BMI, body mass index; ER, estrogen receptor; PARP, poly‐ADP ribose polymerase; RRM, risk reducing mastectomy.

Of those eligible for breast conservation, 13 patients had received genetic test results prior to surgery. Eight of these patients opted for therapeutic mastectomy, five of whom also opted for contralateral risk reducing mastectomy (RRM) with their cancer surgery, and one of whom plans an RRM once their cancer treatment is complete. Five patients opted to continue with breast conservation, two of whom plan to have risk reducing bilateral deep inferior epigastric artery perforator (DIEP) flap reconstruction when their BMI has reduced.

Of those eight patients who did not have genetic test results at the time of surgery, three are planning bilateral risk reducing mastectomies.

### 3.2. Patients Not Eligible for Breast Conservation Breast Cancer Surgery

Five patients with a P/LP variant required a therapeutic mastectomy as they were not eligible for breast conservation. Two patients had a historic therapeutic mastectomy on the contralateral side. One patient had results returned before surgery and declined an RRM. Of the two remaining patients, one has planned an RRM and one is still receiving systematic anticancer therapy and risk reducing surgery has not yet been discussed.

One patient was eligible under R444.1 only. They underwent breast conservation and have currently declined bilateral risk reduction surgery. Two patients were found to have distant metastasis and thus did not have surgery. Overall, 15 of the 28 patients with a P/LP variant have or will likely change their surgical plan based on this positive result (Figure 2).

Three patients with a BRCA pathogenic variant were eligible for treatment with a PARPi. One patient would have been eligible but was diagnosed prior to R444.1 criteria being approved.

## 4. Discussion

“Next‐generation sequencing,” a technology for DNA and RNA sequencing and detection of P/LP variants, has transformed genetic testing, enabling faster, higher‐capacity laboratory workflows [18]. Rather than testing every patient, criteria were developed to identify most P/LP variants or those likely to benefit from PARPi [6, 19]. By identifying variants during the treatment pathway, patients are enabled to make more informed decisions about their surgical and oncological options.

### 4.1. Who Should We Test?

Patients were informed of their eligibility for genetic testing within days of their cancer diagnosis. The timing of the conversation, where the genetic susceptibility is raised, can be challenging. Following a literature review of the psychological impact of genetic testing in breast cancer patients, Schlich‐Bakker et al. suggested that when discussing genetic testing, clinicians need to be aware of possible high psychological distress and additional counseling needs [20]. Identification of a high‐risk P/LP variant can influence surgical options, as well as offer the opportunity for PARPi [19, 21]. However, for some patients, it is simply too early for them to consider this, and it is reasonable to allow for breast conservation with a plan to revisit conversations later. Neoadjuvant chemotherapy may allow for more time and opportunity to discuss testing. We found that there needed to be an individualized approach to consent, and that patients varied in their readiness for testing.

14.3% of our breast cancer patients, eligible for genetic testing, were found to carry a P/LP variant. Although time consuming to accurately determine the criteria for testing under family history scoring, we showed the importance of a robust system to assess eligibility to ensure the pick‐up rate of P/LP variants reaches the 10% minimum target as set out by NICE and Genomics England [7].

The aggregate prevalence of P/LP variants has been estimated at 5%–10% among women with breast cancer [1, 2, 22]. Total incidence of P/LP variants within our breast cancer patient population analyzed during our study period was 1.5%. If we include those patients previously known to have a P/LP variant who developed breast cancer within the time frame and also assume that there was a 14.3% chance of picking up a P/LP variant in those patients who were eligible but not tested, this is increased to 2%. This is significantly fewer than the 5%–10% expected. However, Hu et al. have discussed that these prevalences and associated risks of breast cancer are based on high‐risk women and studies of only limited size have evaluated P/LP variants in multigene panels in women with breast cancer unselected for family history or age at diagnosis [23]. Thus, current estimates of prevalence of P/LP variants may be overcalled. Furthermore, there are increasingly more patients who are known to have a P/LP variant and choose risk reduction surgery before a breast cancer develops. These patients would not appear in the mainstream screening population, as their risk reducing surgery removes the risk of developing breast cancer.

Criteria for testing are a controversial subject. It has been suggested to offer genetic testing to all patients with breast cancer [15]. Tung et al. argue that this would place an enormous strain on the limited number of genetic counselors and that cost‐effectiveness data are inconsistent. They discuss that studies have shown an increase in inappropriate mastectomies, omission of radiation, and even unnecessary oophorectomies for patients with moderate‐risk variants. There is also a lack of a clinical workforce adequately trained in cancer genetics, which increases the potential for misinterpretation of variants and inappropriate management recommendations [24].

The team leading the BRCA‐DIRECT trial are hoping to reduce workforce and costs associated with testing, which would enable testing for all. Torr et al. have developed a digital pathway for diagnostic germline genetic testing and offered testing to all new breast cancer patients using a buccal swab. They demonstrated noninferiority (and superiority) in uptake of testing when utilizing a fully digital pathway vs partially digital pathway. 3% (30/999) of participants were found to have a P/LP variant. What is not clear is how many additional patients were identified that would have been tested through the R208/R444.1 criteria [25].

American Society of Surgical Oncology (ASCO) Guidelines recommend testing patients diagnosed with breast cancer ≤ 65 years. If > 65 years, testing is also available if triple‐negative, a personal, or family history to suggest the possibility of a P/LP variant, patients with breast cancer assigned male at birth, those of Ashkenazi Jewish ancestry or are members of a population with an increased prevalence of founder variants. These criteria although still more complex than testing all would test many more patients than those under the R208/R444.1 criteria [26].

We would argue that one area of resistance to adoption of all R208/R444.1 pathways is the complexity of the eligibility criteria, especially when considering the family history eligibility. To address this, we accelerated our process initially with the use of an electronic patient questionnaire whereby patients entered information online before their clinic appointment. There were direct questions relating to all criteria associated with the Manchester scoring system. Despite this, within our cohort, 22 patients who were eligible were not tested, and as this was a retrospective audit, there was limited information as to why. There may be several reasons including possible communication errors between those determining eligibility and the surgeon seeing the patient. It may not have been clear to the team at the time that these patients were eligible. These issues would be more apparent during the infancy of a pathway implementation when change to a pathway can be a challenge to clinical teams. This should be noted as a potential cause of selection bias. To address this, in collaboration with the computer science department at the University of Bristol, we are developing an algorithmic digital program that will assess eligibility for all patients if appropriate parameters are entered. This runs alongside other codes as part of a general MDT decision making tool looking at eligibility for several MDT outcomes in addition to the R208/R444.1 criteria.

**FIGURE A2:**
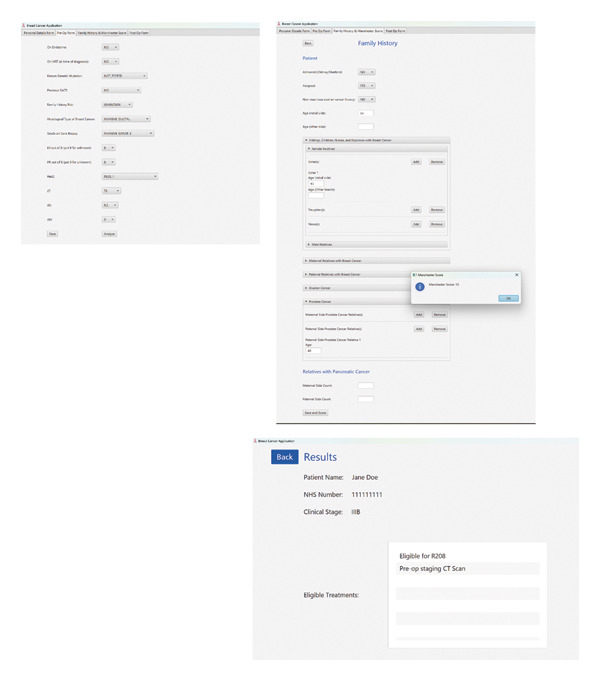
Test patient used as an example of use of algorithmic program developed by the team at RUH, bath. (a) Demonstration of Manchester score based on input of data. (b) Demonstration of preoperative patient information. (c) Results generated by program based on the data submitted—showing eligibility for genetic testing under R208.

Figure A2 demonstrates a patient’s eligibility for R208 using the Manchester score. From data entered, the Manchester score is 15 = 4 (patient age 55) + 6 (sister: cancer breast at 45) −1 (ER positive HER2 negative) + 4 (adopted) + 2 (paternal side prostate cancer at 40). Having reached the threshold of 15, the code allows the clinician information to be generated—in this demonstration case, the patient is eligible for testing under R208.

**FIGURE A3:**
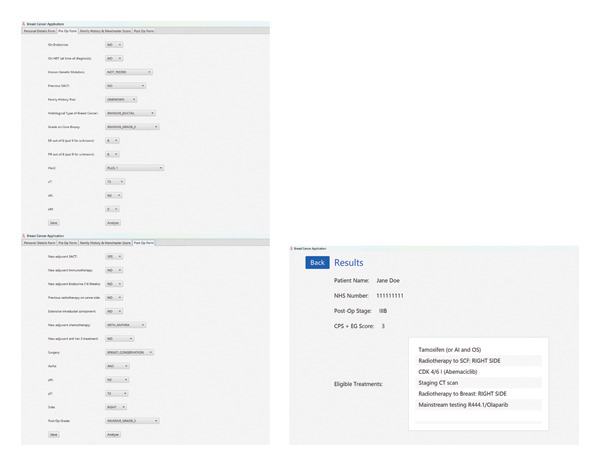
Test patient used as an example of use of algorithmic program developed by the team at RUH, bath. (a) Demonstration of preoperative patient information. (b) Demonstration of postoperative patient information. (c) Results generated by program based on the data submitted—showing eligibility for a number of therapies including eligibility for genetic testing under R444.1.

Figure A3 similarly shows a patient who has an ER positive, HER 2 negative breast cancer who has had neoadjuvant chemotherapy and is eligible for R444.1 as they have a calculated CPS + EG score of 3. This program runs alongside the MDT and thus reveals other treatments and investigations for which this demonstration patient would be eligible.

Having discussed who to test, we realize that a small proportion of patients declined testing at initial consultation. In our cohort, this amounted to 24 patients. It is expected that a few would decline testing as they would prefer not to know a result that would not change their prognosis; the reasons that our patients declined testing were not examined within this study. When interrogating the notes, the main reason given was that they would like to revisit this decision in the future. As an implementation outcome, we will recommend more formal documentation regarding declining of testing. Smith‐Uffen et al. suggest that patients may decline testing due to language barriers, cultural and religious background, poor understanding, education, and literacy [27]. In terms of ethnic background, the population that the RUH serves is predominantly white (88.7%). When looking at the index of multiple deprivation quintile, only 3.4% of our patients are within the most deprived group [28]. While a lower socioeconomic status may be a factor associated with nonconsent for testing, there are likely to be other reasons that would be important to understand in ongoing research.

### 4.2. Surgical Choices

Where positive genetic test results were available and discussed with patients during the initial therapeutic surgical planning, the majority of patients opted for therapeutic mastectomy and, where eligible, contralateral risk reducing surgery (either immediate or delayed). Eligibility for risk reduction surgery for patients deemed to be at high risk was determined by the geneticist at the rapid access clinic. For those patients who were eligible for testing and were considering a bilateral DIEP flap reconstruction, the time frame to have results returned was very tight. This was especially so if there was no indication for neoadjuvant treatment. When an earlier result was imperative, the team at the regional Genomics Hub were very accommodating. It was also important to have the challenging conversation that having a mastectomy or indeed an RRM would not alter the prognosis of their presenting breast cancer [29].

A proportion of patients who had tested positive for a P/LP variant and deemed to be high risk by the genetics team chose breast conservation surgery. They commonly felt that they were not ready for a mastectomy and required more time to process the genetic diagnosis and its implications. There is much discussion about the relevance of risk reducing surgery in patients who have a cancer diagnosis. Copson et al. following the POSH study suggested that decisions about the timing of additional surgery aimed at reducing future second primary‐cancer risks should consider patient prognosis associated with the first malignancy as well as patient preferences. Within this study, of the 338 patients with a BRCA pathogenic variant, 54 were reported to have developed a contralateral breast cancer within the follow‐up period, which may have been prevented had they undergone risk reduction surgery [29]. More recently, Blondeaux et al. have shown that patients with BRCA pathogenic variant, a history of early breast cancer (stages I–III) and those 40 years or under, have a significantly improved overall‐ and disease‐free survival if they undertake risk reducing contralateral mastectomy [30].

Care should be taken when offering mastectomy rather than breast conservation to patients with a P/LP variant, which confers a lower risk than variants such as BRCA. Some patients with such variants, when fully assessed by clinical genetics, may only have a moderate risk of a further breast cancer, for example, some ATM variants. Some women with an ATM have a lifetime risk of breast cancer of approximately 25%, the majority of which are ER‐positive, and it is not yet clear how the identification of an ATM variant in a patient newly diagnosed with breast cancer would impact their treatment and follow‐up [31]. Similarly, patients with a VUS should be advised against risk reduction surgery [32, 33]. This is especially so as there is emerging evidence that there is a benefit in the overall survival in favor of breast conservation surgery over mastectomy for all breast cancer patients [34].

### 4.3. Further Implications of a Positive Test

We have seen that genetic testing, while providing patients with an opportunity to see whether an underlying P/LP variant has contributed to the development of their cancer, can result in anxiety and guilt with respect to the wider family. We cannot underestimate the complexity of the emotions around both a cancer diagnosis and the implications of genetic testing on patients and their families. Individual family dynamics can play a role in how patients feel about testing. Hughes et al. have found that many women found to have a PV go on to inform family members relatively quickly, with sisters often passing on information within a week of the test result [35]. However, it has been noted that communicating results to family members may be a stressor for P/LP variant carriers [36, 37]. With increased access to testing, we must consider the implications for the family members of patients who test positive for a P/LP variant. Furthermore, the more tests we do, the higher the pick‐up for VUS, adding uncertainty for patients and potentially increasing anxiety for patients and their families [20].

In our experience, many patients described their own parents having symptoms of guilt in passing on a defective gene. Additionally, several relatives accessed private testing while waiting for the NHS genetic service. This led to problems with relatives who test positive in the private sector subsequently asking for surgery, before meeting the Clinical Genetics Service. There have been some uncertainties surrounding the validity of some private testing [38, 39]. We have also noted, where several siblings have been found to be positive, there are differing approaches to telling their children. Awareness of these issues has facilitated the counseling process delivered by our cancer support nurse and has helped to prepare patients who are being tested for the challenges that may arise within the family.

For those patients who have a strong family history but do not test positive for a P/LP variant included in the R208/R444.1 panels, a referral for further counseling and testing for further P/LP variants (e.g., Tp53) from the genetics department is arranged. Tp53‐associated cancers usually present at a very young age with a very clear history of this gene within the family [40]. Even if no P/LP variant is identified after further testing, there are other factors that determine risk in families such as single nucleotide polymorphisms and these patients may benefit from a genetic assessment and subsequent management with either surgery or more frequent and prolonged screening [41].

Another important area of discussion is the subject of insurance. The Code of Genetic testing and insurance is an agreement between the Government and the Association of British Insurers (ABI). It explains what an insurance company is entitled to know about the genetic testing a patient may have had when they are accessing insurance. Currently, a patient would not have to tell insurers about the results of a predictive genetic test unless they are buying the largest amounts of insurance compared to the general market (e.g., life insurance above £500,000). This could change [42].

### 4.4. Limitations

Although we demonstrated that the criteria for mainstream genetic testing result in a P/LP variant in > 10% of those tested, there are limitations in this study.

This is a single‐center design in which only a small number of patients were found to harbor a P/LP variant. The local population served makes the data limited in its generalizability to the whole UK population. As we were not testing all patients within the scope of an audit, we are not able to know the total number of patients with a P/LP variant.

Furthermore, there is possible selection bias from patients who both declined testing and from those “not‐discussed” patients. As a department, we did not implement formal notation as to why patients declined testing and as such are unable to undertake a formal analysis of those who declined. Given the difficult circumstances of a cancer diagnosis, some patient refusal is expected; a prospective study to explore reasons patients decline genetic testing would be valuable. Several eligible patients were not tested due to presumed communication errors, clinician omission, or undocumented declines.

While we looked at those patients who were deemed eligible, we did not look for those who may have been eligible but missed. There is a dependence on the patients knowing and communicating their family history. Moreover, those eligible under the more straightforward criteria such as under 40 years old may also have been missed, although less likely. Similarly, eligible patients for the R444.1 may not have been picked up at the MDT.

In the surgical clinic, family history is obtained directly from the patient and assumed to be accurate, rather than reviewing all the relevant patient records: as is the standard practice in a genetics clinic. This reliance on patient‐reported family history and possible miscommunication between relatives regarding ages and cancer types may result in some patients being tested outside true eligibility.

The surgical decisions were made following a referral to the clinical geneticist and discussions with a variety of surgeons. There would be clinician biases and possibly fixed patient preferences, thus resulting in differences when counseling patients in their decision to have breast conservation or mastectomy.

## 5. Conclusion

Access to mainstream genetic counseling and testing for P/LP variants associated with breast cancer has been a successful addition to the service that can be provided within the traditional breast clinic. While time consuming to determine eligibility, we have developed robust methods to speed up this process including the implementation of a preappointment questionnaire and development of an algorithmic program. We continue to improve our counseling skills with both practice and by listening to patient feedback. The criteria set out to pick up ≥ 10% of patients with a P/LP variant are accurate in our population. This has allowed patients to make informed choices in their cancer pathways and changed treatment decisions or pathways for 14 women found to harbor a P/LP variant. We recognize that the wider emotional implications of genetic testing for some patients and their families should not be underestimated.

## Author Contributions

**Soraya Conroy:** formal analysis (lead), visualization (equal), investigation (equal), and writing–original draft preparation (lead); **Emma Keane:** data curation (equal) and writing–review and editing (equal); **Bushra Rehman:** data curation (equal), conceptualization (equal), and writing–original draft preparation (equal); **Eleanor Wyborn:** data curation (equal); **Catherine Brown:** data curation (equal); **Lamb Chen:** software (equal); **Josh Chatten:** software (equal); **Elijah Durston-Rand:** software (equal); **Blaise Sheehan:** software (equal); **Elizabeth Ferguson:** data curation (equal); **Tracie Miles:** writing–review and editing (equal); **Emma Beck:** visualization (equal); **Alison Hunter-Smit**h: formal analysis (equal), visualization (equal), and writing–review and editing (equal); **Christiana Laban:** writing–review and editing (equal); **Katherine Smith:** formal analysis (equal) and writing–review and editing (equal); **Diana Dalgliesh:** formal analysis (equal) and writing–review and editing (equal); **Rebecca Bowen:** formal analysis (equal) and writing–review and editing (equal); **Nicola Laurence:** conceptualization (lead), formal analysis (lead), project administration (lead), investigation (lead), supervision (lead), writing–original draft preparation (equal), and writing–review and editing (equal).

## Funding

No funding was received for this manuscript.

## Conflicts of Interest

The authors declare no conflicts of interest.

## Supporting Information

Additional supporting information can be found online in the Supporting Information section.

## Supporting information


**Supporting Information 1** Supporting Table 1: This table details the specific pathogenic or likely pathogenic variations identified in each individual patient.

## Data Availability

The data that support the findings of this study are available on request from the corresponding author (NJL). The data are not publicly available due to restrictions, e.g., their containing information that could compromise the privacy of research participants.
