# Supplementary material for: Implementation and Audit of Mainstream Genetic Testing Within a High‐Volume UK Breast Unit for Pathogenic Variations Associated With Breast Cancer Using the R208 and R444.1 National Test Directory Criterion
Source: Breast J. 2026 Jul 27;2026:2657384. doi: 10.1155/tbj/2657384 (PMC13402888; doi:10.1155/tbj/2657384)
Supplement: Supplementary file 1 — Supporting Information 1 Supporting Table 1: This table details the specific pathogenic or likely pathogenic variations identified in each individual patient. [file TBJ-2026-2657384-s001.docx]

**Supplementary material**

**Supplementary Table 1 : Pathogenic Variations Identified**

(HGVS = Human Genome Variation Society)

| Pathogenic Variant | HGVS description | Location | Classification |
| --- | --- | --- | --- |
| BRCA1 | NM_007294.3:c5309G>tp.(Gly1770Val) | Chr17:g.41203103G>T | Likely pathogenic |
| BRCA1 | NM_007294.3:c.68_69del_p.(Glu23Valfs) | Chr17(GRCh37):g41276045_41276046del | Pathogenic |
| BRCA1 | NM_007294.3:c.5153G>A p.(Trp1718*) | Chr17:41215390c>T | Pathogenic |
| BRCA1 | Deletion of exons 2-23( traditional |  | Pathogenic |
| BRCA1 | NM_007294.3:exon 20-23 deletion |  | Pathogenic |
| BRCA1 | NM_007294.3:c.181T>G p.(Cys61Gly) | Ch17(GRCh37):g.41258504A>C | Pathogenic |
| BRCA1 | NM_007294.3:c.1501_1504delp.(Lys501*) | Chr17(GRCh37:g41246048_41246051del | Pathogenic |
| BRCA1 | NM_007294.3:c.679G>T p.(GLu227*) | Chr17:41246869C>A | Pathogenic |
| BRCA2 | NM_000059.3:c.658 659del p.(Val220llefs*4) | Chr13(GRCh37):g.32903606 32903507del | Pathogenic |
| BRCA2 | NM_000059.3:c1257del p.(Cys419Trpfs*11) | Chr13(GRCh37):g.32906872del | Pathogenic |
| BRCA2 | NM_000059.3:c6275_6276del p.(Leu2092Profs*7) | Chr13(GRCh37):g32914767_32914768del | Pathogenic |
| BRCA2 | NM_000059.3:c.7988A>t p.(GLu2663Val) | Chr13(GRCh37):g32937327A>T | Pathogenic |
| BRCA2 | NM_000059.3:c.7993del p.(Asp2665llefs*8) | Chr13(GRCh37):g32937332del | Pathogenic |
| PALB2 | NM_024675.3:c.599del p.(Leu200*) | Chr16(GrCh37):g.23647271del | Pathogenic |
| PALB2 | no detailed information as tested by local genetics on stored DNA |  | Pathogenic |
| PALB2 | NM_024675.3:c.2325dup p.(Phe776llefs*26) | Chr16(GrCh37):g.23641150dup | Pathogenic |
| PALB2 | NM_024675.3:c.3350G>A p.? | Chr16(GrCh37):g.23619185C>T | Pathogenic |
| CHEK2 | NM_007194.3:c1100del p.(Thr367Metfs*15) | Chr22(GRCh37):g.29091857del | Pathogenic |
| CHEK2 | NM_007194.3:c1100del p.(Thr367Metfs*15) | Chr22(GRCh37):g.29091857del | Pathogenic |
| CHEK2 | NM_007194.3:c1100del p.(Thr367Metfs*15) | Chr22(GRCh37):g.29091857del | Pathogenic |
| CHEK2 | NM_007194.3:c1100del p.(Thr367Metfs*15) | Chr22(GRCh37):g.29091857del | Pathogenic |
| CHEK2 | NM_007194.3:c1100del p.(Thr367Metfs*15) | Chr22(GRCh37):g.29091857del | Pathogenic |
| CHEK2 | NM_007194.3:c1100del p.(Thr367Metfs*15) | Chr22(GRCh37):g.29091857del | Pathogenic |
| CHEK2 | NM_007194.3:c1100del p.(Thr367Metfs*15) | Chr22(GRCh37):g.29091857del | Pathogenic |
| CHEK2 | NM_007194.3:c1100del p.(Thr367Metfs*15) | Chr22(GRCh37):g.29091857del | Pathogenic |
| ATM | Deletion of exon 9 |  | Likely Pathogenic |
| RAD51D | NM_002878.4:c667+13del p.? | Chr17(GRCh37):g.33430471 33430473del | Likely Pathogenic |
